# Molecular characterization of a new emaravirus infecting *Clerodendrum thomsoniae* plants

**DOI:** 10.1007/s00705-026-06734-x

**Published:** 2026-09-05

**Authors:** Vinicius Henrique Bello, Pedro Luis Ramos-González, Gianluca L. Michea Gonzalez, Fernanda de Pádua Del Corona, Jorge Alberto Marques Rezende, Elliot Watanabe Kitajima

**Affiliations:** 1 Universidade de São Paulo/Esalq - Depto. de Fitopatologia e Nematologia, Av. Pádua Dias 11, Piracicaba, 13418-900 Brazil; 2 Laboratório de Biologia Molecular Aplicada, Instituto Biológico, São Paulo, Brazil

## Abstract

High-throughput sequencing revealed a new emaravirus named clerodendrum yellow blotch virus (ClYBV), infecting *Clerodendrum thomsoniae* plants in São Paulo State, Brazil. Four genomic RNA segments (RNA1, RNA3, RNA4, and RNA5) were recovered, whereas RNA2 remained undetected. Phylogenetic analysis and pairwise comparisons based on amino acid sequences showed ClYBV is most closely related to redbud yellow ringspot-associated virus (RYRaV, *Emaravirus cercidis*). No ClYBV particles were observed by transmission electron microscopy in leaf tissues from infected plants. These results support the identification of ClYBV as a new *Emaravirus* member, though the undetectable RNA2 suggests the isolate may be defective, lacking this segment and unable to produce virus particles. The binomial species name “*Emaravirus clerodendri*” is proposed.

**Supplementary Information:**

The online version contains supplementary material available at https://doi.org/10.1007/s00705-026-06734-x.

Viruses of the genus *Emaravirus* belong to the family *Fimoviridae* (order *Bunyavirales*) and are transmitted by eriophyoid mites. Emaraviruses have a segmented single-stranded, negative-sense RNA genome, and their virions are membrane-bound particles approximately 100 nm in diameter [1]. The number of genomic segments varies between species, from four to ten. The four main genomic segments encode the RNA-dependent RNA polymerase (RdRP, RNA1) essential for RNA replication; a glycoprotein precursor (GP, RNA2) likely involved in virus-mite interactions; the nucleocapsid protein (NP, RNA3) involved in the encapsidation of RNA; and the movement protein (MP, RNA4) related to viral movement [1–3].

Emaraviruses cause a variety of systemic plant diseases, frequently associated with mosaics, chlorotic ringspots, yellow blotches, leaf deformation, and chlorosis [3]. To date, members of 33 species, including *Emaravirus actinidiae*,* Emaravirus artemisiae*,* Emaravirus cajani*,* Emaravirus camelliae*,* Emaravirus cercidis*,* Emaravirus sorbi* and *Emaravirus rosae*, have been reported to infect cultivated and ornamental plants in different regions of the world (https://ictv.global/taxonomy).

In 2017, two glory-bower (*Clerodendrum thomsoniae* Balf.f.) plants showing yellow blotch symptoms were collected in a residential garden in the municipality of Piracicaba, São Paulo State, Brazil [4]. Cuttings of these plants were planted and kept in a glasshouse at the Escola Superior de Agricultura Luiz de Queiroz, University of São Paulo (Fig. 1a). Total RNA was individually extracted from two symptomatic leaves of a single plant using the RNeasy Plant Mini Kit (QIAGEN, Venlo, the Netherlands). For each sample, a cDNA library was prepared using the Ribo-Zero Kit for plants (Illumina, San Diego, USA) to remove ribosomal RNA, and the libraries were subsequently sequenced using high-throughput sequencing (HTS) on an Illumina NovaSeq6000 system (2 × 100-bp paired-end reads, Macrogen Inc., Seoul, Korea).

**Fig. 1:**
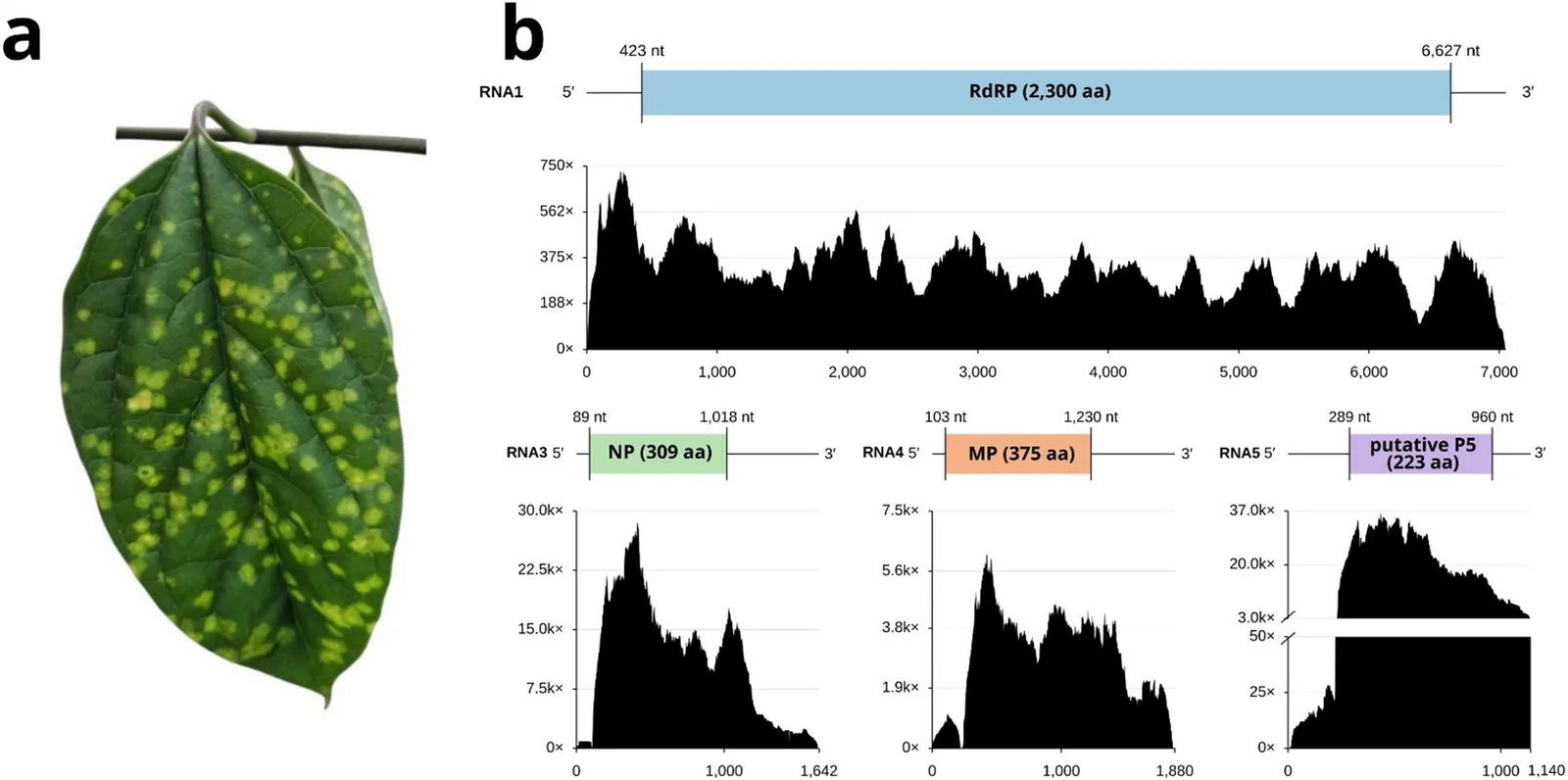
(**a**) Symptoms of yellow blotches on leaves of a *Clerodendrum thomsoniae* plant. (**b**) Schematic representation of the genomic organization of clerodendrum yellow blotch virus (ClYBV). Open reading frames of each RNA are represented as gray boxes, nt (nucleotide), aa (amino acid), RdRP (RNA-dependent RNA polymerase), NP (nucleocapsid protein), MP (movement protein), and putative P5 (putative protein 5)

The HTS reads were trimmed and assembled de novo using SPAdes v3.15.3 and Trinity v2.15.1, both implemented on the Galaxy platform 23.2.rc1 (https://usegalaxy.org/). To identify plant virus-related contigs, BLASTn and BLASTp programs available on the Galaxy platform were employed using databases containing plant viral genomes obtained from NCBI Virus (https://www.ncbi.nlm.nih.gov/labs/virus/vssi/#/). HTS of the two samples generated more than 30 million reads (Supplementary Table S1). From the two libraries assembled from the clean reads, four contigs containing emaravirus-related sequences were detected. BLAST analysis also allowed the identification of two other contigs corresponding to Clerodendrum leaf spot virus (CLSV, *Dichorhavirus piracicabense*), a virus previously characterized [4]. CLSV, like other dichorhaviruses, causes nonsystemic diseases under natural conditions [5].

The four contigs, ranging in length from 1,513 to 7,058 nucleotides (nt) and presumably representing segments of an emaravirus, were consistently recovered from the libraries of both samples. The cognate pairs showed identity values higher than 99%. Therefore, consensus sequences were generated for each pair of corresponding contigs and used for the characterization of the viral genome. In each contig, a single open reading frame (ORF) was identified (Fig. 1b), and based on the highest nucleotide identity values with ORFs found in typical members of the genus *Emaravirus*, they were designated as RNA1, 3, 4, and 5 of a tentative new virus called clerodendrum yellow blotch virus (ClYBV) [3, 6].

RNA 1 (PZ229612) is the largest genomic fragment containing 7,058 nt, including a 44-nt 5’ untranslated region (UTR) and a 111-nt 3’ UTR. This segment contains a large ORF encoding a 2,300-amino-acid (aa) RdRP with an estimated molecular mass of 267 kDa. The conserved Bunyavirus RdRP domain (pfam04196) was identified in this protein using the NCBI Conserved Domain Database (CDD) [7]. RNA 3 (PZ229613) is the smallest genome fragment containing 1,513 nt and includes an 88-nt 5’ UTR and a 495-nt 3’ UTR, and the ORF on it encodes the NP (309 aa, 34 kDa). RNA 4 (PZ229614) comprises 1645 nt with a 102-nt 5’ UTR and a 418-nt 3’ UTR, and the identified ORF codes for the MP (375 aa, 42 kDa), in which the characteristic viral P4 movement protein domain of emaraviruses (pfam16505) was identified using the NCBI CDD. RNA 5 (PZ229615) contains 1,527 nt, including a 288-nt 5’ UTR and 567-nt 3’ UTR. It comprises one ORF that encodes a putative protein designated P5 (223 aa, 25 kDa). This putative protein also occurs in at least 30 other emaraviruses, including those of the species *Emaravirus actinidae*, *Emaravirus cercidis*, and *Emaravirus rosae*. The first 13 nt of the 5′-termini (RNA1 and 4: AGUAGUGAACUCC, RNA3: GGUAGUGAACUCC, RNA5: CCCUUCCAGUUCU) and the last 13 nt of the 3′-termini (RNA1: GGAGUUCUCUACU, RNA3: AAUUUCCAGUUGC RNA4, and 5: GGAGAACACUACU) in the RNA segments of this virus were similar to those in other emaraviruses [1, 8]. The implementation of the UTR-backed iterative BLASTn approach [9] using conserved nucleotide sequences of ClYBV RNAs resulted in the recovery of contigs corresponding to the already known segments, and no new putative genomic RNAs were identified.

Sequence alignments of deduced amino acids of each ClYBV protein were performed using ClustalW implemented in Geneious Prime 2026.1.1 (Dotimatics). ClYBV proteins showed the highest sequence identities of 72.60%, 62.10%, 63.04%, and 48.43% to those of redbud yellow ringspot-associated virus (RYRSaV, *Emaravirus cercidis*), respectively (Supplementary table S2). Maximum-likelihood phylogenetic trees based on the amino acid sequences of the RdRP, NP, MP, and P5 were constructed using IQ-Tree with automatic model selection and 1,000 ultra-fast bootstrap replicates (CIPRES Science Gateway). Across all four phylogenetic analyses, ClYBV grouped within four well-supported clades, containing RYRSaV, lilac chlrotic ringspot-associated virus (LiCRaV, *Emaravirus syringae*), actinidia chlorotic ringspot-associated virus (AcCRaV, *Emaravirus actinidiae*) and european mountain ash ringspot-associated virus (EMARaV, *Emaravirus sorbi*) (Fig. 2). However, in the MP- and P5-based phylogenetic trees, EMARaV clustered in clades different from those containing ClYBV (Fig. 2). This phylogenetic pattern is consistent with previous studies showing that members of the genus *Emaravirus* are generally classified into four major phylogenetic clades for each protein [8, 10, 11]. Recombination analysis using RDP5 v5.67 did not find evidence of interspecies recombination (data not shown).

**Fig. 2:**
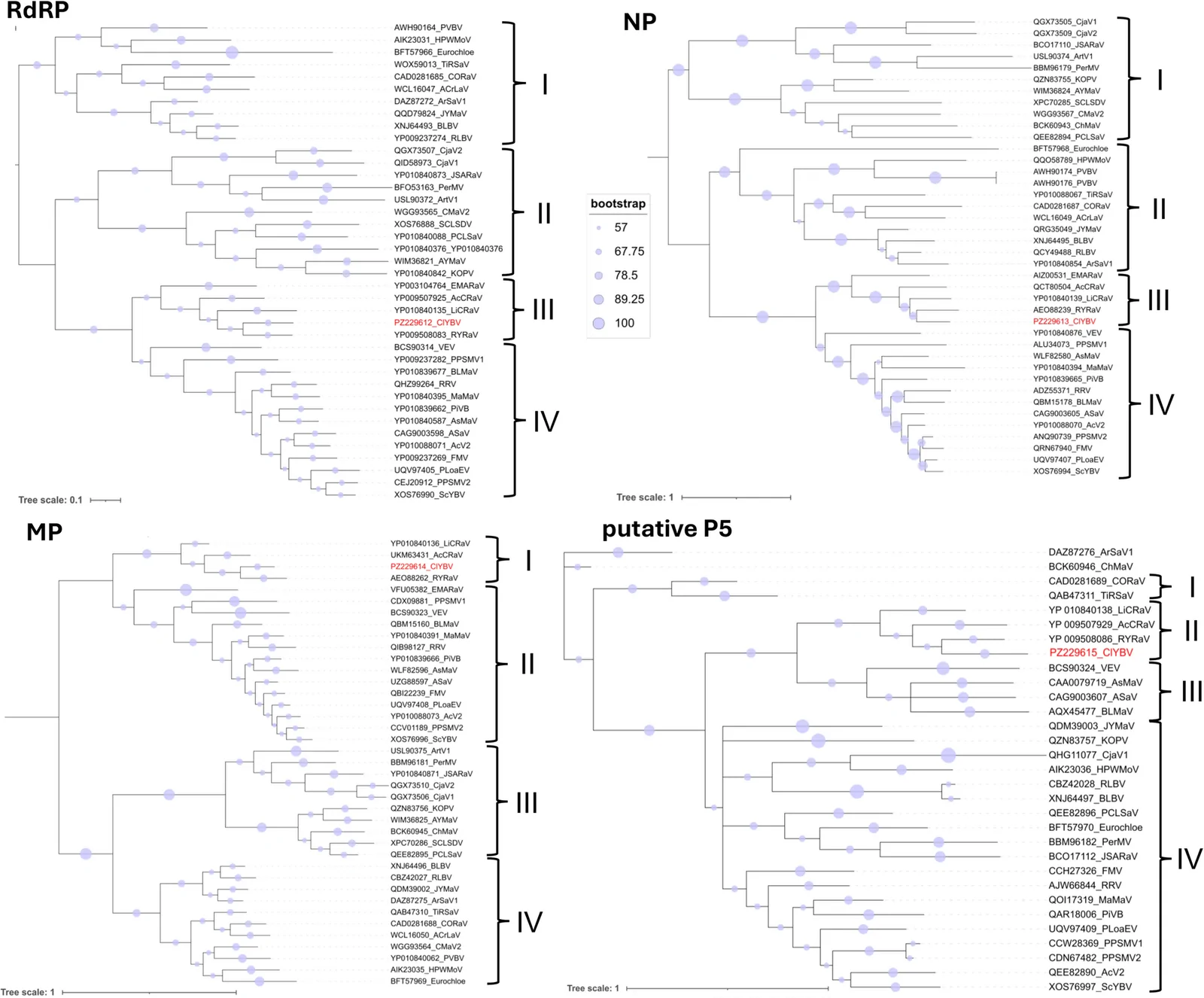
(**a**) Maximum likelihood trees inferred from multiple alignments of amino acid sequences of the RdRP, NP, MP, and putative P5 of clerodendrum yellow blotch virus (ClYBV) and other emaraviruses sequences obtained from GenBank. The final trees were visualized, rooted and edited using the iTOL software. Amino acid sequences of this work are highlighted in red. The acronyms and full names of the viruses, as well as the respective species, are available in supplementary Table 2

To validate the HTS results and provide a diagnostic method, the specific primers ClYBV_RdRP_2998/3017 (ATAGTCGTGGTGAGGTAGAA) and ClYBV_RdRP_3412/3393 (GTACCACCAGCTCTATCAAG) (TM = 55 °C) were designed using Geneious Prime. The emaravirus RNA1 was detected via RT-PCR from the total RNA of both plants. A single band corresponding to an amplicon of 415 bp corresponding to RdRP region was obtained.

To detect presumptive ClYBV particles, symptomatic leaves of *C. thomsoniae* plants were examined by transmission electron microscopy (TEM). Small leaf pieces were embedded in Spurr’s low-viscosity epoxy resin and sectioned with a LEICA UC6 ultramicrotome. Ultrathin tissue sections were collected on 300-mesh copper grids, stained with 3% uranyl acetate followed by Reynolds’ lead citrate, and examined with a JEOL JEM-1011 transmission electron microscope [12]. Emaravirus-like particles were not observed in the ultrathin sections. However, dense masses were found in some epidermal and parenchymal cells from the lesion’s tissues (Fig. 3), which may correspond to aggregates of non-enveloped viral nucleoprotein. Similar electron-dense masses have also been reported in plants infected with defective isolates of tomato spotted wilt virus (TSWV, *Orthotospovirus tomatomaculae*) [13].

**Fig. 3:**
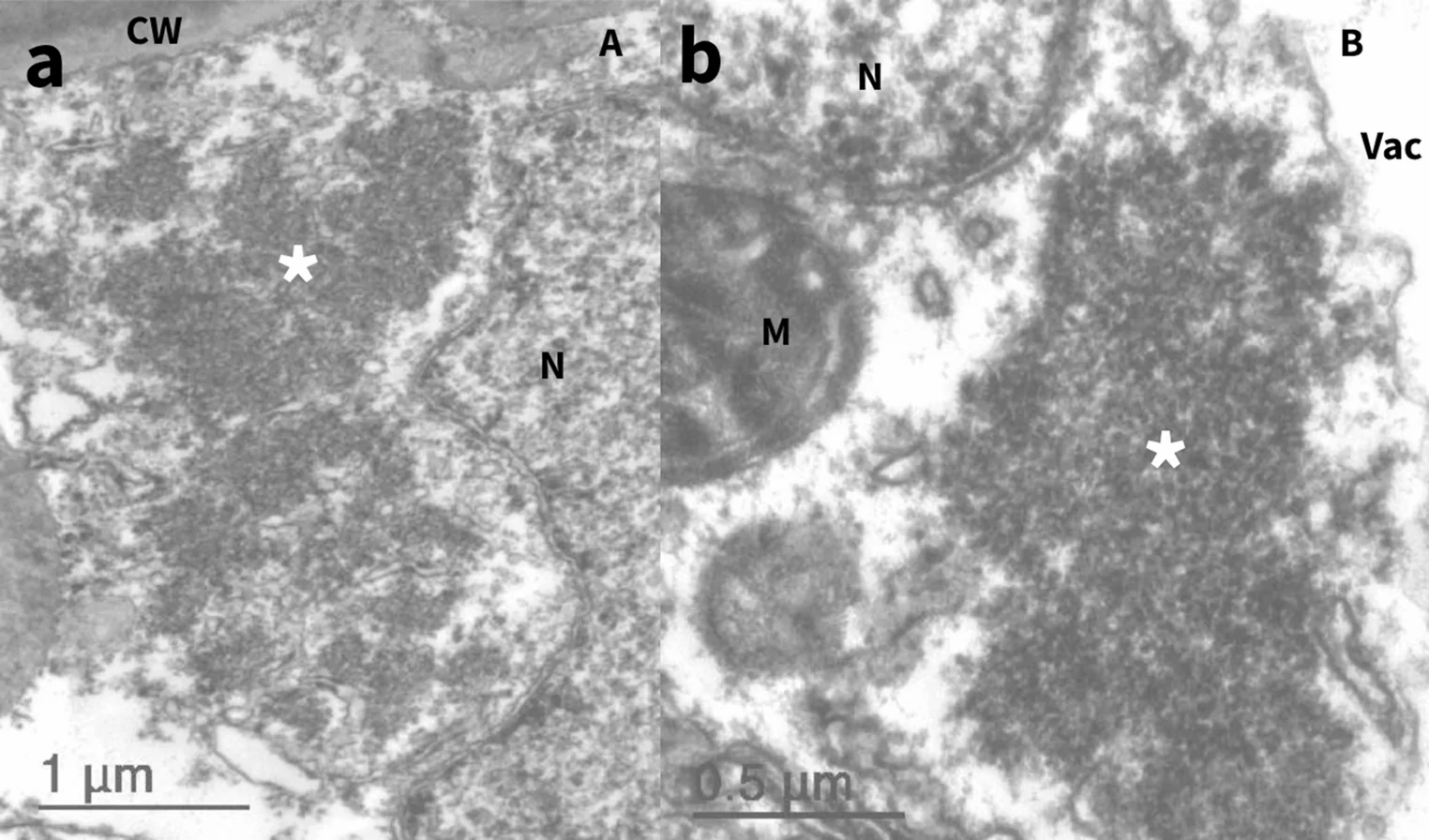
Transmission electron micrograph of sections of glory-bower (*Clerodendrum thomsoniae)* leaf chlorotic spots revealing the presence of electron dense masses (*) in the cytoplasm, respectively of parenchymal (**a**) and epidermal (**b**) cells. CW- cell wall; M- mitochondrion; N- nucleus; Vac- vacuole

The absence of emaravirus-like particles in the examined ultrathin sections may be attributed to the undetectability of RNA2 in this isolate. The absence of RNA2 was previously observed in five of the 21 isolates of perilla mosaic virus (PerMV, *Emaravirus perrilae)* from Japan analysed at the molecular level [2]. Furthermore, an experimental study demonstrated that an infectious clone of rose rosette virus (RRV, *Emaravirus rosae*) formed solely of RNA1, RNA3, RNA4, and RNA5 was able to infect *Nicotiana benthamiana* plants [14]. However, the complete genomes of PerMV and RRV include RNA2. Instead, the absence of RNA2 in ClYBV raises the possibility that the characterized isolate represents a defective form.

ClYBV was transmitted to two uninfected *C. thomsoniae* plants (out of a total of 2) by grafting, and the original yellow blotch symptoms were reproduced 60 days after grafting. Viral infection was confirmed by RT-PCR using specific primers as previously described. There is no information on a possible vector. Neither symptoms nor RNAs from the dichorhavirus CLSV were detected in the infected plants.

Members of different species in the genus *Emaravirus* are distinguished based on the amino acid sequence identity of the gene products encoded by RNA1 (RdRP), RNA2 (GP), RNA3 (NP) and RNA4 (MP). Emaraviruses sharing less than 80% of aa sequence identity of at least two deduced proteins must be considered members of different species (https://ictv.global/report/chapter/fimoviridae/fimoviridae/emaravirus). Data from this study clearly demonstrate that ClYBV is a new species in the genus *Emaravirus.* However, since RNA2 was not detected, the characterized isolate might perhaps be considered a potentially defective form lacking the RNA2 segment, rather than a complete representation of the ClYBV genome. Furthermore, this is the first emaravirus described infecting *Clerodendrum thomsoniae* in Brazil.

## Supplementary Information

Below is the link to the electronic supplementary material.

Supplementary Material 1

Supplementary Material 2

## Data Availability

No datasets were generated or analysed during the current study.
