# Supplementary material for: Molecular characterization of a new emaravirus infecting Clerodendrum thomsoniae plants
Source: Arch Virol. 2026 Sep 5;171(10):271. doi: 10.1007/s00705-026-06734-x (PMC13546163; doi:10.1007/s00705-026-06734-x)
Supplement: Supplementary file 1 [file 705_2026_6734_MOESM1_ESM.docx]

**Supplementary table 1.** Library composition, viral genome coverage, and generated contigs corresponding to the genome of clerodendrum yellow blotch virus (ClYBV) from two datasets obtained via high-throughput sequencing from an infected *Clerodendrum thomsoniae* plant.

| Total of reads | 61,706,190 | | | | 70,159,792 | | | |
| --- | --- | --- | --- | --- | --- | --- | --- | --- |
| Dataset | 1 | | | | 2 | | | |
| Emaravirus fragment length (nt) | RNA1 7,050 | RNA3 1,642 | RNA4 1,880 | RNA5 1,140 | RNA1 7,048 | RNA3 1,532 | RNA4 1,646 | RNA5 1,379 |
| Viral RNA matched reads | 23,347 | 184,056 | 55,839 | 192,670 | 328,103 | 1,505,310 | 1,363,362 | 1,272,084 |
| Mean coverage of viral bases | 333.65x | 11,292.38x | 2,991.26x | 16,970.46x | 4,690.70x | 98,913.15x | 83,449.29x | 92,806.26x |
| Viral genome recovery (%) | 99.93 | 100 | 99.36 | 99.12 | 100 | 99.02 | 99.94 | 98.98 |
| Number of generated contigs (length of the contig) | 1 (7,050) | 1 (1,642) | 1 (1,880) | 1 (1,140) | 1 (7,048) | 1 (1,532) | 1 (1,646) | 1 (1,379) |
