# Supplementary material for: Molecular characterization of a new emaravirus infecting Clerodendrum thomsoniae plants
Source: Arch Virol. 2026 Sep 5;171(10):271. doi: 10.1007/s00705-026-06734-x (PMC13546163; doi:10.1007/s00705-026-06734-x)
Supplement: Supplementary file 2 [file 705_2026_6734_MOESM2_ESM.docx]

**Supplementary Table 1.** Percentage of pairwise amino acid sequence identities for the proteins coded by RdRP, N, MP, and putative P5 of clerodendrum yellow blotch virus (ClYBV) and 38 other emaraviruses.

| **Emaravirus*** | **RdRP** | | **NP** | | **MP** | | **putative P5** | | |
| --- | --- | --- | --- | --- | --- | --- | --- | --- | --- |
|  | **GenBank access codes** | **Percentage** | **GenBank access codes** | **Percentage** | **GenBank access codes** | **Percentage** | | **GenBank access codes** | **Percentage** |
| JSARaV | YP010840873 | 29,70% | BCO17110 | 25,68% | YP_010840871 | 18.48% | | BCO17112 | 6.61% |
| KOPV | YP010840842 | 29,80% | QZN83755 | 25,23% | QZN83756 | 17.03% | | QZN83757 | 7.01% |
| AsMaV | YP010840587 | 48,00% | WLF82580 | 38,00% | WLF82596 | 31.60% | | CAA0079719 | 17.61% |
| MaMaV | YP010840395 | 48,00% | YP010840394 | 34,70% | YP_010840391 | 33.22% | | QOI17319 | 14.86% |
| ChMaV | YP010840376 | 29,80% | BCK60943 | 29,15% | BCK60945 | 15.01% | | BCK60946 | 11.50% |
| LiCRaV | YP010840135 | 62,80% | YP010840139 | 61,80% | YP_010840136 | 56.35% | | YP_010840138 | 35.87% |
| PCLSaV | YP010840088 | 29,50% | QEE82894 | 25,59% | QEE82895 | 16.93% | | QEE82896 | 6.50% |
| BLMaV | YP010839677 | 47,70% | QBM15178 | 36,00% | QBM15160 | 32.37% | | AQX45477 | 10,42% |
| PiVB | YP010839662 | 48,50% | YP010839665 | 37,30% | YP_010839666 | 34.43 | | QAR18006 | 10.00% |
| AcV2 | YP010088071 | 48,40% | YP010088070 | 39,10% | YP_010088073 | 36.68% | | QEE82890 | 11.24% |
| RYRaV | YP009508083 | 72,60% | AEO88239 | 62,10% | AEO88262 | 63.04% | | YP_009508086 | 48.43% |
| AcCRaV | YP009507925 | 65,50% | QCT80504 | 58,10% | UKM63431 | 56.19% | | YP_009507929 | 35.87% |
| PPSMV1 | YP009237282 | 48,80% | ALU34073 | 34,90% | CDX09881 | 31.39% | | CCW28369 | 11.50% |
| RLBV | YP009237274 | 34,10% | QCY49488 | 21,20% | CBZ42027 | 23.93% | | CBZ42028 | 9.62% |
| FMV | YP009237269 | 47,80% | QRN67940 | 40,70% | QBI22239 | 34,94% | | CCH27326 | 8.27% |
| EMARaV | YP003104764 | 55,10% | AIZ00531 | 40,80% | VFU05382 | 35.71% | | - |  |
| ScYBV**** | XOS76990 | 48,90% | XOS76994 | 38,40% | XOS76996 | 33.30% | | XOS76997 | 11.01% |
| SCLSDV**** | XOS76888 | 27,60% | XPC70285 | 28,93% | XPC70286 | 16.44% | | - |  |
| BLBV**** | XNJ64493 | 33,70% | XNJ64495 | 26,42% | XNJ64496 | 17.49% | | XNJ64497 | 9.52% |
| AYMaV | WIM36821 | 29,70% | WIM36824 | 20,70% | WIM36825 | 19.01% | | - |  |
| CMaV2**** | WGG93565 | 29,00% | WGG93567 | 29,45% | WGG93564 | 23.12% | | - |  |
| ArtV1 | USL90372 | 27,10% | USL90374 | 25,00% | USL90375 | 24.62% | | - |  |
| PLoaEV | UQV97405 | 47,80% | UQV97407 | 38,40% | UQV97408 | 34.62% | | UQV97409 | 12.62% |
| JYMaV | QQD79824 | 34,20% | QRG35049 | 18,90% | QDM39002 | 22.71% | | QDM39003 | 6.00% |
| CjaV1 | QID58973 | 28,30% | QGX73505 | 27,87% | QGX73506 | 21.25% | | QHG11077 | 9.88% |
| RRV | QHZ99264 | 47,90% | ADZ55371 | 34,00% | QIB98127 | 21.6% | | AJW66844 | 12.97% |
| PPSMV2 | CEJ20912 | 48,20% | ANQ90739 | 39,10% | CCV01189 | 23.87% | | CDN67482 | 12.31% |
| ASaV | CAG9003598 | 48,90% | CAG9003605 | 42,31% | UZG88597 | 32.83% | | CAG9003607 | 16.71% |
| *Emaravirus urochloe*** | BFT57966 | 27,00% | BFT57968 | 18,70% | BFT57969 | 23.25% | | BFT57970 | 9.12% |
| PerMV | BFO53163 | 27,90% | BBM96179 | 31,52% | BBM96181 | 16.77% | | BBM96182 | 8.76% |
| VEV | BCS90314 | 48,40% | YP010840876 | 36,70% | BCS90323 | 28.48% | | BCS90324 | 17.06% |
| ACrLaV | WCL16047 | 34,04% | WCL16049 | 18,50% | WCL16050 | 21.06 | | - |  |
| TiRSaV | WOX59013 | 42,10% | YP010088067 | 21,70% | QAB47310 | 22.16% | | QAB47311 | 8.19% |
| PVBV | AWH90164 | 36,51% | AWH90174 | 25,76% | YP_010840062 | 23.30% | | - |  |
| CORaV | CAD0281685 | 37.39% | CAD0281687 | 28,87% | CAD0281688 | 18.10% | | CAD0281689 | 9.17% |
| HPWMoV | AIK23031 | 35.2% | QQO58789 | 22,30% | AIK23035 | 15.17% | | AIK23036 | 11.46% |
| CjaV2 | QGX73507 | 31.67% | QGX73509 | 25,97% | QGX73510 | 15.07% | | - |  |
| ArSaV1 | DAZ87272 | 35.56% | YP010840854 | 22,90% | DAZ87275 | 16.14% | | DAZ87276 | 11.87% |

*AcCRaV (actinidia chlorotic ringspot-associated virus, *Emaravirus actinidiae*), ACrLaV (ailanthus crinkle leaf-associated virus, *Emaravirus ailanthi)*, ArtV1 (artemisia fimovirus 1, *Emaravirus artemisiae*), PPSMV1 (pigeonpea sterility mosaic virus 1, *Emaravirus cajani*), CjaV1 (camellia japonica-associated virus 1, *Emaravirus camelliae*), RYRaV (redbud yellow ringspot-associated virus, *Emaravirus cercidis*), ChMaV (chrysanthemum mosaic-associated virus, *Emaravirus chrysanthemi*), AYMaV (clematis yellow mottle-associated virus, *Emaravirus clematis*), TiRSaV (ti ringspot-associated virus, *Emaravirus cordylinae*), KOPV (karaka Ōkahu purepure emaravirus, *Emaravirus corynocarpi*), FMV (fig mosaic virus, *Emaravirus fici*), ASaV (ash shoestring-associated virus, *Emaravirus fraxini*), RLBV (rasberry leaf blotch virus, *Emaravirus idaeobati*), JSARaV (japanese star anise ringspot-associated virus, *Emaravirus illicii*), AcV2 (actinidia virus 2, *Emaravirus kiwii*), PLoaEV (pueraria lobata-associated emaravirus, *Emaravirus kudzu*), PVBV (palo verde broom virus, *Emaravirus parkinsoniae*), PerMV (perilla mosaic virus, *Emaravirus perillae*), PiVB (pistacia virus B, *Emaravirus pistaciae*), AsMaV (aspen mosaic-associated virus, *Emaravirus populi*), PCLSaV (pear chlorotic leaf apot-associated virus, *Emaravirus pyri*), CORaV (common oak ringspot-associated virus, *Emaravirus quercus*), RRV (rose rosette virus, *Emaravirus rosae*), BLMaV (Blackberry leaf mottle-associated virus, *Emaravirus rubi*), EMARaV (european mountain ash ringspot-associated virus, *Emaravirus sorbi*), LiCRaV (lilac chlrotic ringspot-associated virus, *Emaravirus syringae*), PPSMV2 (pigeonpea sterility mosaic virus 2, *Emaravirus toordali*), HPWMoV (high plains wheat mosaic virus, *Emaravirus tritici*), CjaV2 (camellia japonica-associated virus 2, *Emaravirus verbanni*), ArSaV1 (arceuthobium sichunense-associated virus 1, *Emaravirus visci*), VEV (vitis emaravirus, *Emaravirus vitis*), JYMaV (jujube yellow mottle-associated virus, *Emaravirus ziziphi*), CMaV2 (callicarpa mosaic-associated virus 2), BLBV (blackberry leaf blotch virus), SCLSDV (spiraea chlorotic leaf spot distortion virus), ScYBV (scaevola yellow blotch virus), MaMaV (maple mottle-associated virus). **tentative species that have not yet been approved by International Committee on Taxonomy of Viruses (ICTV); - Virus species without RNA 5.
